# Perceptions toward a pilot project on blended learning in Malaysian family medicine postgraduate training: a qualitative study

**DOI:** 10.1186/s12909-018-1315-y

**Published:** 2018-08-29

**Authors:** Hani Salim, Ping Yein Lee, Sazlina Shariff Ghazali, Siew Mooi Ching, Hanifatiyah Ali, Nurainul Hana Shamsuddin, Maliza Mawardi, Puteri Shanaz Jahn Kassim, Dayangku Hayaty Awang Dzulkarnain

**Affiliations:** Department of Family Medicine, Faculty of Medicine & Health Sciences, Universiti Putra Malaysia, 43400 UPM Serdang, Selangor Malaysia

**Keywords:** Continuing medical education, Postgraduate training, Family medicine, Blended learning, Qualitative research methods

## Abstract

**Background:**

Blended learning (BL) is a learning innovation that applies the concept of face-to-face learning and online learning. However, examples of these innovations are still limited in the teaching of postgraduate education within the field of family medicine. Malaysian postgraduate clinical training, is an in-service training experience and face-to-face teaching with the faculty members can be challenging. Given this, we took the opportunity to apply BL in their training. This study provides an exploration of the perceptions of the educators and students toward the implementation of BL.

**Methods:**

A qualitative approach was employed using focus group discussions (FGD) and in-depth interviews (IDI) at an academic centre that trains family physicians. Twelve trainees, all of whom were in their hospital specialty’s rotations and five faculty members were purposively selected. Three FGDs among the trainees, one FGD and two IDIs among the faculty members were conducted using a semi-structured topic guide. Data were collected through audio-recorded interviews, transcribed verbatim and checked for accuracy. A thematic approach was used to analyse the data.

**Results:**

There were four main themes that emerged from the analysis. Both educators and trainees bill the perspective that BL encouraged continuity in learning. They agreed that BL bridges the gap in student-teacher interactions. Although educators perceived that BL is in concordance with trainees learning style, trainees felt differently about this. Some educators and trainees perceived BL to be an extra burden in teaching and learning.

**Conclusion:**

This study highlights a mix positive and negative perceptions of BL by educators and trainees. BL were perceived positively for continuity in learning and student-teacher interaction. However, educator and learner have mismatched perception of learning style. BL was also perceived to cause extra burden to both educators and learners. Integrating BL to a traditional learning curriculum is still a challenge. By knowing the strengths of BL in this setting, family medicine trainees in Malaysia can use it to enhance their current learning experience. Future study can investigate different pedagogical designs that suit family medicine trainees and educators in promoting independent learning in postgraduate training.

## Background

Medical education has undergone various changes over the past decades and in recent years, the adoption of technologies in teaching and learning are becoming popular in many higher institutions around the world, to include Blended Learning (BL). BL is defined as the thoughtful fusion of face-to-face and online learning experiences [1]. Examples of these innovations in family medicine postgraduate teachings are still limited and the majority of innovations are found primarily in undergraduate teaching [2–6].

In a busy clinical setting, continuous learning using BL approach is a useful way to gain and keep up with ever-growing medical knowledge. The face-to-face component of BL is a way of transferring knowledge into practice in primary care settings [7]. Primary care practitioners often deal with disease that presents itself at an early stage. Often these problems are undifferentiated and unorganized in manner. A systematic review of reports, although with limited evidence, indicates that blended learning has the potential to improve clinical competencies, such as clinical reasoning, history taking and reflective thinking skills [7]. It is important for primary care practitioners to be able to deal with the uncertainties with updated knowledge and through it be able to eventually engage in the right clinical reasoning and decision-making skills to arrive at the correct diagnosis or course of action.

Blended learning has positive impacts on knowledge and satisfaction among users. A study done by Bösner et al. found that BL that uses the inverted classroom approach has significant impact on overall gain in skills and knowledge in the teaching of differential diagnosis in primary care among the medical students [2]. The undergraduate medical students involved in that study perceived this approach to learning very positively in terms of satisfaction of the teaching content, flexibility of time and acquisition of teamwork [2]. Another study done by Lewin et al. found that online curricula using the BL model for primary care preceptorship was rated very highly by medical students [3]. The rating was excellent for module content particularly related to charting and direct patient care. Students who participated in this program had high performance scores for history taking, physical exam, medical knowledge and patient education as compared to the study’s control group [3]. Other disciplines that implemented BL in undergraduate medical teachings included orthopaedics, nursing and pharmacy, which all showed similar results [4, 6, 8].

BL allows learners to learn independently at their own time of choice and allows theory to be translated into practice, which is an important characteristic for clinicians who are trained while they are on the job [9, 10]. Because the delivery of blended learning relies on the mixture of face-to-face and online learning environments, students can benefit from increased time and spatial flexibility for their study, wider and easier access to learning resources, and a higher level of autonomy in regulating their learning [10]. BL allows practice to be incorporated more holistically into the learning pathway by including practice-based assignments [11, 12].

This study aimed to engage in an exploration of the perceptions of educators and trainees in the Master of Family Medicine training through implementation of a project using BL in their teaching and learning activities. This teaching method in postgraduate training is of relevance to educators and students in many medical disciplines and provides insight in improving the training of medical postgraduates in family medicine.

As this is a qualitative study, it can help to generate hypotheses to be tested in larger sample. We hope to initiate a more structured, innovative pedagogy model in the teaching of family medicine in the future. The feedback provided by the educators and trainees to identify challenges and facilitators are valuable in implementing BL in family medicine postgraduate training and to inform future intervention in teaching and learning.

## Methods

As mentioned above, this study employs a qualitative study approach to explore the perceptions of educators and trainees regarding blended learning. If dissemination and use of the study’s results is sufficiently impactful, this study will be the first phase of a mixed methods approach to understanding the impact of blended learning in a medical postgraduate training centre.

### Setting

Family medicine is one of the departments in a public university situated in the central region of Malaysia. The department offers the teaching of Family Medicine to undergraduate Bachelor of Medicine and postgraduate Masters of Family Medicine students.

#### The master of medicine (family medicine) program

The Master of Medicine (Family Medicine) is a four-year in-service clinical training program. It is aim to produce competent family physician who will provide specialise care at primary health care centres throughout Malaysia. In the first 2 years of the program, the trainees will undergo hospital-based rotations i.e. medicine, obstetrics, orthopaedic, at accredited hospitals for postgraduate training under the Ministry of Health throughout Malaysia. Professional exam (I) will take place at the end of year one. In the existing curricula, trainees will only have formal face-to-face learning sessions twice a year, where they will attend the teachings in-campus. These teachings will be facilitated by the faculty members who are practicing family medicine specialists. Other learning opportunities will be at their respective hospitals, where they are based, and by the appointed clinical supervisors, who are not a member of the faculty. The clinical supervisors are selected based on their expertise in the respective fields.

In year three and year four of the training, trainees will be posted at primary health care centres to continue their training. The trainees will attend weekly face-to-face teaching at the faculty. Professional exam (II) will take place at the end of year three. In year four, trainees are to complete a research project in their area of interest. Professional exam (III) consists of submission of a thesis dissertation, a family case report and a clinical practice diary. They need to pass all these components in order to be awarded the Master of Medicine (Family Medicine).

### Method

In 2016, the department incorporated BL as part of the department’s teaching and learning method for Family Medicine postgraduate trainees. Prior to this implementation, educators underwent training on the concept of BL and types of online applications that can be used to supplement BL activities.

We started a six-month project using blended learning approach to enrich the learning experience of the first and second year trainees who are based outside the faculty throughout the academic year. A virtual learning component was added to the existing face-to-face teaching sessions. The educators in the department were involved in the designing of the added online component, taking into account the blended learning goals outlined by Osguthorpe et al. [13]. The idea of this added component is to develop better understanding of learning goals among the trainees, to improve interactions between students and educators, to promote active and continuous learning and to improve ease of revision using cost effective methods. The engagement with the family medicine specialist throughout the hospital rotations provides a platform for reflections among the trainees about their experience in the hospital. The students were briefed about the BL activities implemented prior to the project.

#### The family medicine Postgraduate’s blended learning project

A free online board was used as a platform for educators to post packages of scenarios on a specific topic i.e. Month 1: Paediatric; Group 1- facial oedema. Each educator prepared and posted a case scenario (including images) based on a given topic. A guide on the problem to be covered were provided to the educators to prevent overlap. This guide is based on the consensus between the educators regarding the common problems seen or diagnoses that should not be missed in a primary care setting. After a satisfactory discussion was done on the initial posted problem, follow-up problems were posted by the educators until the trainees reached a correct diagnosis and were able to formulate a satisfactory management plan. The discussion included differential diagnosis to the presenting problem, important history and examinations, options for investigations and a holistic management approach. Each learning group consisted of an educator and two trainees. Both educators and trainees completed these tasks at own time, but time given for one discussion was 1 month. At the end of the discussion, the other group had access to another group’s discussion and discussed the cases that they had among themselves.

During these exercises, trainees were able to discuss their experience managing these cases in the hospital setting and were encourage to reflect on these experiences as primary care doctors. At the seventh month of using this new blended learning approach, trainees and educators were invited to be interviewed to explore their experiences using BL.

### Participants

The study took place in a small family medicine unit. Thus, we employ universal sampling method for this study. There are two groups of participants in this study; postgraduate trainee and educator.

All trainees who are in their first and second year of training and educators who were involved in the project were invited through email invitations to participate in the interview. The email was send by a research assistant to protect anonymity of participants. After a week, when there was no response from the emails, mobile message invitation was sent out. Three mobile reminders were sent out and the participants responded positively. Participation was solely voluntary. Those who replied to the email or the mobile messages were provided the details of the study and the participants’ information sheet.

All twelve trainees and five educators agreed to participate in the interviews. One educator declined to be interviewed. Trainees were allowed to form their own discussion groups. Groups discussion allows interaction between members and rich information can be obtained in such discussions [14]. One postgraduate trainee was being interviewed in an IDI, mainly due to logistic issue. The interviews took place in the faculty. Participants were paid an honorarium after they completed the interview to compensate them for their time and effort.

### Data collection

The interviews were carried out using a semi-structured topic guide for educators (Table 1) and for trainees (Table 2). The theoretical framework for the topic guide incorporated the constructs of Social Cognitive Theory, which addresses behaviour as a dynamic process that involves interaction between the person, behaviour and the environment [15]. Behaviour change, even as part of learning process, is more likely when a person believes in his or her own capacity to change (self-efficacy) and values the outcome expectancies as a result of their personal action. This topic guide was piloted among three trainees outside this project’s unit and amendments were made based on consensus among the researchers.

**Table 1:** Semi-structured interview guide for educator

I am going to ask you a series of questions, please respond to the following questions based on your experience:	
ᅟ1. What do you understand by the term blended learning? Give examples ᅟ2. Could you describe your last experience with blended learning? ᅟ3. How do you feel about using blended learning? ᅟ4. How does blended learning differ from the usual traditional classroom meetings/teaching? ᅟ5. What are your aims when you apply blended learning in your teachings? ᅟ6. How does the blended learning assist your teaching process? ᅟ7. What are the difficulties you had in using blended learning in the course of your teaching? ᅟ8. You recently used the online application in your teaching, what will motivate you to continue using it? ᅟ9. Any difficulties using it? ᅟ10. Can you share with us the things that facilitate you in using the online application? ᅟ11. How equipped are you in using blended learning?

**Table 2:** Semi-structured interview guide for trainees

I am going to ask you a series of questions, please respond to the following questions based on your experience:	
ᅟ1. Could you describe your last experience with the combination of online learning with your usual face-to-face teachings? ᅟ2. How do you feel about this new learning approach? ᅟ3. How does this new learning approach assist your learning process? ᅟ4. What are the difficulties you had during this new learning approach in this course of learning? ᅟ5. What motivates you to use this approach in the future? ᅟ6. Can you share with us the things that facilitate you in using this new learning approach?	

This study used face-to-face focus group discussions (FGD) and in-depth interviews (IDI) as an attempt to triangulate the data by collecting the data through two types of interview methods. In-depth interview is a highly effective method to collect rich data [14]. It allows discussion of the issues they faced and other experiences that are not necessarily evident to researcher. In-depth interview will also allow probing of sensitive topics, if any, brought up by participants. Group discussion is also employed as it allows participants to engage in many and important forms of everyday communication, verbal and non-verbal, such as jokes and arguments, that would not be captured in quantitative data collection [14]. In group discussions, information will relatively quick to generate as collective of opinions will be gathered from more than one person in a single session, compared to individual interviews [14].

The interviews (FGD and IDI) were conducted by a bilingual, independent and experienced moderator (LYK) from outside the faculty who is not known to the participants prior to this study. Each interview was carried out in the faculty meeting room. The interviews were conducted in a conversational format using Malay or English, as preferred by the participants. Choice of language was left to the participant is to allow, without reservation, exploration of perceptions, thoughts, feelings and experiences of the participants about BL. An assistant moderator (NM) was present to aid in taking detailed notes on the order of speakers, made observations about the discussion, and recorded field notes including facial expressions, comments and interpersonal interactions that occurred during the FGDs. The format of the interviews (FGD and IDI) were selected based on convenience. On average, the FGDs lasted between 1 to 1.5 h and IDIs lasted between 30 to 45 min.

All the interviews were audio recorded, transcribed verbatim and translated from Malay language with all respondent’s identifiers anonymised. To ensure anonymity, audio recordings were handled by the assistant moderator whereby identifiers in the recording were deleted. The audio-recordings were then transcribed by a transcriber outside the faculty and further identifiers were deleted. There were no repeat or follow-up interviews.

### Data analysis

#### Reflexivity

In this qualitative study, the researcher is the research tool who is directly involved in the research process. Thus, self-reflection is essential to avoid past experience or preconceived ideas or roles outside the study from influencing the research process, particularly in the thematic coding and interpretation of interview data.

In line with this exercise, we acknowledge that the researchers are educators who are directly involved with the development and preparation of the contents of the project. We may favour blended learning and expect positive outcomes from the trainees but we constantly reminded ourselves that different learners may learn differently from what we consider to be the ideal learning style. The environment was very much in a clinical setting, and as family doctors, we tailored our treatment plans according to the needs of our patients. We approached our study subjects similar to the needs of our patients in regard to blended learning. In carrying out this study, we gathered strategies from its proponents to improve the delivery of blended learning. We also agreed that the negative voices critical of blended learning are the voices of the unmet needs that we can take on and use to improve our way of teaching.

As educators and the researchers in this study, there is a potential conflict of interest in the educator-trainee relationship. Efforts were made throughout the study to eliminate identifiers by selecting a focus group and interview moderator from outside the faculty, handling of audio-recordings and transcribing them without allowing identification of respondents based upon voice characteristics. Measures to reduce any undue influence was the assurance given to the participants to ensure unbiased opinions from the trainees and the educators during the interview and to ensure impartial analyses by the researchers. Although the research team members were not involved in the transcribing process due to the potential bias mentioned, the researchers immersed themselves in the data by iterative reading of data during the data analysis phase.

#### Transcribing

A trained transcriber, who was independent of the study, transcribed and checked all the interviews. All the recordings were transcribed in the spoken language. The emotional context and non-verbal communication were included as notes in the transcripts. Word-for-word translations of the conversations from Malay to English were performed during the data analysis. Contextual meaning was used to produce a meaning-based translation where literal translation could not convey the intended meaning of the participants [16]. The data were organized using QSR NVivo 10 qualitative data analysis software (Nvivo Qualitative Data Analysis Software).

#### Thematic analysis

Thematic analysis was conducted following the six phases described by Braun and Clarke [17]. These phases include “1) familiarizing with the data, 2) generating initial codes, 3) searching for themes, 4) reviewing themes, 5) defining and naming themes, and 6) producing the report.” Inductive thematic analysis was conducted in view of a lack of previous studies done in this area. The analysis involved coding the entire data set and the codes were collated to identify themes [14]. The emerging themes were combined into overarching themes and grouped into categories [14].

The data analysis started during the data collection itself and this is done iteratively. We conducted the focus groups on different days and after each focus group, the interviews were transcribed and translated immediately. Two groups of researchers consisted of five researchers: Group 1(LPY, HS), Group 2 (SSG, HF, CSM) and each group coded the transcripts separately and comparison of findings was done thereafter. Discussion was conducted between the researcher groups to reach to a consensus. This allowed us to review the transcripts through rereading and familiarizing with the data to identify initial codes. We reviewed the interview from the initial group, to either confirm or refute the emerging codes during subsequent focus groups [14].

### Ethical issues

Ethical appraisal of the study plan was obtained from the Ethics Committee of the university (FPSK (EXP15) P187). In accordance with the ethical appraisal, written informed consent to participate in the interviews and to publish non-identifiable information in journal publications was obtained from participants prior to the interviews.

## Results

In total, three FGDs and three IDIs were conducted among the educators and trainees. Two FGDs and one IDI were carried out among the trainees. In this setting, there are more female trainees and educators, a common demographic in a Family Medicine unit in this region. One FGD and two IDIs were carried out among the educators. Both the IDI and FGD were used to collect data as to confirm the emerging findings. Each FGD consist of 3 to 5 participants.

### Characteristics of participants

In total, there are seventeen participants with age ranges from 32 to 50 years-old. Characteristics of the participants are summarised in Table 3. There were 12 trainees and five educators.

**Table 3:** Summary of participant characteristics

Interview	Participants	Age	Gender
FGD 1	Educator 1	39	Female
	Educator 2	50	Female
	Educator 3	37	Female
IDI 1	Educator 4	40	Female
IDI 2	Educator 5	38	Female
FGD 2	Trainee 1	34	Female
	Trainee 2	32	Female
	Trainee 3	34	Female
	Trainee 4	32	Female
	Trainee 5	33	Female
	Trainee 6	32	Male
FGD 3	Trainee 7	32	Female
	Trainee 8	33	Female
	Trainee 9	32	Female
	Trainee 10	32	Female
	Trainee 11	36	Male
IDI 3	Trainee 12	34	Female

### Summary of themes

For both educator’s and trainee’ s interviews, four main themes emerged in the analysis. Table 4 summarise the themes expressed by the participants.

**Table 4:** Summary of themes expressed by participants

Interview	Participants	Themes			
		TH1	TH2	TH3	TH4
FGD 1	Educator 1	x		x	
	Educator 2		x	x	x
	Educator 3	x	x	x	
IDI 1	Educator 4			x	x
IDI 2	Educator 5	x	x		x
FGD 2	Trainee 1	x	x	x	
	Trainee 2	x		x	
	Trainee 3	x		x	
	Trainee 4	x	x		
	Trainee 5		x	x	x
	Trainee 6		x		x
FGD 3	Trainee 7			x	x
	Trainee 8	x			x
	Trainee 9	x	x		
	Trainee 10		x	x	
	Trainee 11			x	
IDI 3	Trainee 12		x	x	x

Note: Theme I: BL encourages continuous learning (TH1)

Theme II: BL bridges the gap in student-teacher interactions (TH2)

Theme III: The different perceptions of teaching and learning styles (TH3)

Theme IV: Perceived BL as an extra burden (TH4)

### Theme I: BL encourages continuous learning

Both educators and trainees expressed that implementation of BL encouraged continuity of learning.

#### Educators

An educator commented that virtual discussion helps trainees to discuss different cases and their approaches to those cases with their respective online supervisor throughout their clinical attachment at their own time. This can spark further discussion during face-to-face discussions either with the educators or their colleagues, which creates continuity in learning. Educators felt that BL promotes continuity in learning and help prepare trainees, who are busy clinicians, for their professional exams.

Another educator pointed out that virtual discussion of BL allows trainees to refer back to their previous discussions on the online applications to find answers for their clinical queries.

*“They can still use the materials discussed virtually beyond the classroom. They can open it in the classroom for the discussion and relook later. It promotes continuity in learning. So, they can go home and reflect on their lesson.” Educator 4, 40 years-old, FGD*

#### Trainees

During the interview with the trainees, some commented that BL encouraged them to read and look up answers before attempting to answer questions posed by lecturers. This improved their knowledge on the discussed topic. One trainee shared that this approach was different in face-to-face interactions when they need to provide spontaneous/immediate responses without having the opportunity to first look for the evidence-based approach before providing an answer. Another participant shared the feeling of embarrassment when the wrong responses were given.

*“It is quite good [BL exercise] because we can revise before we answer all the questions. [This method] improves our knowledge in approaching certain disease. Unlike in class, if I were to answer on the spot a given question and it’s wrong, it is shameful” Trainee 9, 32 years-old, FGD*

However, many felt that BL helped to prepare them for the theory part of the professional exams but not the clinical exams. When asked for a reason for this opinion, some felt that clinical teachings must be done face-to-face in the presence of patients.

BL encourages many who participate in this project to continue their learning at own time. One trainee pointed out that BL is a driving force for them to continuously learn.

*“[BL] forces us to learn because when we are given a scenario, we come up with history, physical examination and then the differential diagnosis. If we are not very sure, we can go back to our books. It forces us indirectly to learn.” Trainee 3, 34 years-old, FGD*

### Theme II: BL bridges the gap in student-teacher interactions

#### Educators

Educators feel that BL serve as a platform that can improve interactions with trainees. Discussion can be made with the trainees regardless of time and place and interactions can become more flexible with BL. An educator said:

“*BL is very good. With our busy schedule we can use the learning applications to connect to the student anytime, anywhere. Yeah, interactions [are] very flexible with BL.” Educator 3, 37 years-old, FGD*

#### Trainees

Similarly, with the trainees, they also felt that BL encouraged fostering trainee-educator interactions. They see this opportunity as a support outside the classroom and felt that this flexibility to be a source of motivation in their post-graduate training. One stated:

*“There’s interaction between students and the lecturer in [BL] approach. It’s good and it’s quite motivating to be able to have more frequent discussions with the lecturer.” Trainee 6, 32 years-old, FGD*

#### Educators and trainees

Both students and educators suggested the use of social media as a platform to be used in this blended learning approach. Especially for educators, not only are they very familiar with the use of social media, it also provides notification and instant feedback. An educator explained:

*“We should also use social media and discussion will be even better. There’s notification, so you know the students has replied. We do not need to relearn to use social media as we have been using it” Educator 2, 50 years-old, FGD*

### Theme III: The different perceptions of teaching and learning styles

#### Educators

Some educators felt that BL can only be used in teaching of technical subjects i.e. Information Technology (IT) and Mathematics. They felt that exclusively face-to face teachings are suitable in medical teaching and superior because it involved human interactions. One said:

*“In technical subject like IT, you probably can apply this learning approach. However, in medicine, it is more of a human subject in which face-to-face interaction is very important and more superior.” Educator 1, 39 years-old, FGD*

Educators shared that the incorporation of virtual learning on top of the trainee’s usual face-to-face teaching was in line with the students’ way of learning because they are the generation that is into gadgets and the internet. One commented that the trainees, being in the Y generation, they are technology-competent and enthusiastic in learning content that is internet-based.

*“There are students who [are] enthusiastic. They responded in a timely manner [for the online exercise]. Probably this is their style of learning. I mean internet-based or technology based. I can see that from their responses.” Educator 5, 38 years-old, IDI*

#### Trainees

Most trainees still preferred traditional face-to-face learning. They felt that this is their way of learning since the undergraduate days, thus, adapting to change at their age will be difficult as postgraduate trainees. However, some trainees agreed that they may need to pick up and adapt a new learning style with the advancement of technology. A trainee explained:

“*We prefer traditional method like face-to-face style. Because we can learn directly from our lecturer. The main reason is that from undergraduate level, our learning had always been face-to-face. That’s how we learnt. At this age, a change is difficult” Trainee 11, 36 years-old, FGD*

Some trainees felt that discussions done virtually lacked the conversational manner and human interactions. They agreed that in medical teaching, discussions need to be real time. Trainees have the perception that BL is like answering an exam-based questions without instant feedbacks. A trainee described:

“*So, example, let say our colleague answer a clinical question, immediately we can clarify and asked them to justify their answers. “Why did you answer this? Why not that?” Then the lecturer can add on her opinions. “You know, I think what she says is right because of....” It will be more like a two-way learning conversation instead of like the one we do online. [BL] has no instant feedback.” Trainee 1, 34 years-old, IDI*

### Theme IV: Perceived BL as an extra burden

#### Educators

In some instances, both educators and trainees felt that the introduction of BL activities to their current teaching and learning is a burden. For educators, they need to prepare the cases to be discussed with the students and had to attend courses for getting familiar with the online learning platform. An educator commented that they may have other commitments such as administrative work, hence, the time taken to learn and prepare online materials can be a burden due to time constraints.

*“We have administrative work that we can’t deny. Now, we need to prepare online exercise for the students to do. It is time consuming.” Educator 2, 50 years-old, IDI*

#### Trainees

As for trainees, juggling between online assignments and daily clinical duties were stressful. Some trainees expressed their frustrations as they felt that BL was just an extra burden. A trainee stated:

“*But this [online exercise] is an extra work to do after work. More like an extra burden!” Trainee 10, 32 years-old, FGD*

## Discussion

We aimed to provide an exploration of perceptions on the implementation of BL in the teaching and learning of postgraduate training for family medicine. The findings of this study contribute to better understanding of the perceptions of both trainees and educators who used BL. Our findings showed agreement and disagreement of negative and positive perceptions among the trainees and the educators with regard to blended approach.

### Continuous learning

Both educators and trainees agreed that BL promotes continuity in learning. This aspect in BL had successful impacts in terms of overall gain in skills and knowledge in clinical reasoning. Literatures reported similar findings, which also included better grades among students in medical training [2, 3, 7]. Online exercises were also found to enhance face-to-face discussions in this study, which is similar with the findings in the literature [5]. A future, structured outcome study can be done to determine the impact of implementation of BL in a postgraduate course and its long-term impact, as suggested in a review on the role of BL on clinical education [7]. It is worth considering a future challenge of looking at the impact of BL based education on the patients’ health outcomes.

It is interesting to note that trainees were favourable of BL because they had the opportunity to prepare their answer to the questions given by their supervisor. This insight is compatible with a study that describes the attitude of Asian students that tend to be nonverbal in class and rarely volunteered or initiated discussions [18, 19]. It is believed that reticence has high regard in Asian culture and that they fear losing face if their answers are wrong [18–20].

### Bridging gaps

Another success of implementing BL in this setting was that it bridged the gap in term of interactions between educators and students. As clinical sites for training can vary geographically for the trainees in our setting, the implementation of blended learning was timely. The online aspect of BL in this project provides an opportunity for trainees to have more interactions with their educators. Studies show that online components of BL improve interaction and provide quality discussion during face-to face meetings [6, 8, 21]. The use of social media was recommended by both educators and students to facilitate interactions. Social media has increasingly been integrated in the higher education learning experience and has been shown to stimulate student’s engagement and collaborative work [11, 22]. Frequent interactions and communications boosted student’s motivation in this study, which were similar to findings in literature [5, 7, 9].

### Learning styles

Educators and trainees had a mismatch of perceptions regarding preferred learning styles. Educators assumed that technology-based learning suited the trainees’ style. However, trainees felt differently about this. As adult learners, learning new technology can be a burden. The difficulty to learn new technologies among adult learners are in keeping with the literature [9]. Successful implementation of BL must take into account learners’ readiness for independent learning and transparent communications regarding the new expectation to use BL is needed from educators [22, 23]. This is to ensure mutual expectations around the new learning environment can be reached between the trainees and the educators. However, it is worth noting that, technology-enhanced learning has the potential to improve self-efficacy and reflective thinking among students in clinical education [7].

### Burdensome

It was interesting to note in this study that educators agreed with the trainees on how learning new technology could be burdensome. On top of that, educators were expected to prepare materials for these blended sessions. The impact on educator’s workload must be considered. It is suggested that shareable and reusable digital teaching materials can help to sustain the use of BL in teaching and learning [24].

Although educators and trainees in this setting found it difficult to adapt their teaching and learning styles with BL, there is evidence from the literature about successful implementation of BL in medical teaching [2, 5, 8]. The pedagogical framework and tools may differ from one another, but the examples can inform a better BL design in the future. However, most implementations are at the undergraduate level. Implementation of BL and its design are still scarce in family medicine postgraduate training. A review summarised that to ensure success, medical pedagogy must be introduced in a scholarly manner and should be a transformative redesign process that rebuilds the course rather than simply adding on technology [23]. Future BL design for postgraduate family medicine training needs to be more structured and be integrated into the curriculum rather than just an added-on technology. The needs of educators and trainees must be considered from time to time to improve the BL design and its sustainability, including the elements that influence attitude toward modern teaching and learning.

### Strengths of this study

One of the strength of this study is the study design whereby the exploration gives a better understanding on the perceptions of both the educators and the trainees on their views and readiness towards BL both the positive and negative perspectives. This knowledge will to inform us on the steps to be done to improve trainees’ learning experience. Another strength is that the trial application of BL in a Malaysian Family Medicine setting.

### Limitations and recommendations

This study did not apply methodological frameworks in qualitative research, but employed qualitative approaches such as interviews. Other limitations of this study include the small sample of students and educators and also the nature of a qualitative study design that the findings may not be fully generalised to other settings. Because this was a project conducted in a single setting, the numbers of participants were limited. Data saturation may not be reached because of this. However, IDI and FGD were employed and multiple investigators were involved to ensure rigour in the emerging findings. Using universal sampling method, we acknowledged its potential bias. One educator who declined the interview might have different views on BL which we were not able to capture. For the participants, we tried to minimise the bias in expressing their views by employing a trained qualitative moderator from outside the faculty. Some of the researchers were also involved in the teaching process but biases were overcome by excluding them from data coding and analysis. Checking of transcripts by the participants was not done because of the potential to breach anonymity due to the small size of the unit and due to the potential conflict of interest in the educator-student relationship outside the study period. Engagement of students in virtual and the subsequent face-to-face discussions were not quantitatively analysed. With that in mind, we can quantify trainees’ engagement to this new approach over a period of time and we can use the findings from this study to assess the feedback by the participants quantitatively in future study design.

In this setting, because educators and trainees were very familiar with social media, it can be considered to be a platform for teaching and learning in the future.

## Conclusion

This study highlights a mix positive and negative perception of BL by educators and trainees. BL were perceived to encourages continuity in learning among trainees and fosters better quality in student-teacher interactions. Nonetheless, adapting BL to conventional and preferred learning is still a challenge as educator and learner have mismatched perception of learning style and BL perceived to cause extra burden to both educators and learners. This will require a redesign of the conventional pedagogical framework for medical students from undergraduate to postgraduate level. Future study can look into implementing BL for a larger respondent sample after considering the findings from this study.

## Abbreviations

BL: Blended learning
FGD: Focus group discussion
IDI: In-depth interview
